# Epidemiological trends of antibiotic resistance in pathogenic *Escherichia coli* in swine farms from the Northwest Iberian Peninsula and evaluation of air sampling for antibiotic resistance surveillance

**DOI:** 10.1186/s40813-025-00475-0

**Published:** 2025-12-29

**Authors:** Gonzalo López-Lorenzo, Adriana Silva, José Manuel Díaz-Cao, Vanessa Silva, Alberto Prieto, Cynthia López-Novo, Gilberto Igrejas, Patrícia Poeta, Gonzalo Fernández

**Affiliations:** 1 Department of Animal Pathology (INVESAGA Group), Faculty of Veterinary Sciences, Universidade de Santiago de Compostela, Campus Terra, Lugo, 27002 Spain; 2 Department of Veterinary Sciences, University of Trás-os-Montes and Alto Douro (UTAD), Vila Real, Portugal; 3 Associated Laboratory for Green Chemistry (LAQV-REQUIMTE), University NOVA of Lisboa, Lisboa, Portugal; 4 IBADER, Instituto de Biodiversidade Agraria e Desenvolvemento Rural, Lugo, Spain; 5 Department of Genetics and Biotechnology, University of Trás-os-Montes and Alto Douro (UTAD), Vila Real, 5000-801 Portugal; 6 Microbiology and Antibiotic Resistance Team (MicroART), Department of Veterinary Sciences, University of Trás-os-Montes and Alto Douro (UTAD), Vila Real, 5000-801 Portugal; 7 Associate Laboratory for Animal and Veterinary Science (AL4AnimalS), University of Trás-os-Montes and Alto Douro (UTAD), Vila Real, 5000-801 Portugal; 8 CECAV—Veterinary and Animal Research Centre, University of Trás-os-Montes and Alto Douro, Vila Real, 5000-801 Portugal

**Keywords:** Air sampling, *E. coli*, Swine, Antibiotic, Resistance, Gene, PCR, Postweaning diarrhea

## Abstract

**Background:**

Monitoring antibiotic resistance levels is essential to preventing its spread in pig farms. This is usually done through tests to assess phenotypic and genotypic resistance, although air analysis in farms could also be useful for obtaining this information. This study examined genotypic and phenotypic resistance levels in pathogenic *E. coli* from pig farms from Northwest of Iberian Peninsula and investigated the potential of air sampling as a tool for estimating antibiotic resistance. Rectal swabs were collected from weaned piglets, along with air samples from their housing areas. Bacteria with potential to cause diarrhea were identified from the swabs, followed by phenotypic antibiotic susceptibility testing and screening for resistance genes targeting eight antibiotic families. The presence of these genes was also analyzed in air samples using qPCR.

**Results:**

All isolated pathogenic *E. coli* exhibited phenotypic resistance to at least two families of antibiotics, with aminoglycosides and lipopeptides being the families to which the highest percentage of resistant *E. coli* was detected (*p* < 0.05). In terms of genotypic resistance, genes encoding resistance to aminoglycosides, penicillins, and cephalosporins were the most frequently detected (*p* < 0.05), whereas those associated with lipopeptides, quinolones, and carbapenems were the least common (*p* < 0.05). Both phenotypic and genotypic resistance tended to cluster according to the origin farm, although positive associations between resistance genes were observed, with *tet*(A) and *bla*_CTX−universal_ being the most frequently associated with others. Regarding air samples, there was poor concordance between the detection of resistance genes in air and the levels of genotypic and phenotypic resistance on the farm (k < 0.4), although this seems to be influenced by the different detection patterns of the evaluated genes in air.

**Conclusions:**

Pathogenic *E. coli* from pig farms exhibited a significant level of phenotypic and genotypic resistance to antibiotics, with resistance to aminoglycosides being particularly notable. Additionally, there was a prominent co-occurrence of genes encoding resistance to multiple antibiotic families. The effectiveness of air sampling for estimating farm-level antibiotic resistance seems to be influenced by differences in gene detection performance in this type of sample.

**Supplementary Information:**

The online version contains supplementary material available at 10.1186/s40813-025-00475-0.

## Background

Postweaning diarrhea (PWD) are one of the major challenges in swine production. Bacteria such as *Escherichia coli*, *Salmonella* spp., *Klebsiella pneumoniae* and others can act as primary agent of the disease (postweaning colibacillosis, edema disease, enteric or septicemic salmonellosis) or as secondary agents in viral infections (rotavirus, transmissible gastroenteritis virus, porcine epidemic diarrhea virus) [1–4]. The dehydration subsequent to PWD may be fatal and compromise piglet growth, thereby necessitating the use of antibiotics to reduce the associated morbidity and mortality [5–9]. Consequently, in many instances affected piglets must be treated empirically until culture and antimicrobial susceptibility results are available.

The use of antibiotics for the treatment of sick animals is a significant contributor to the emergence of antibiotic-resistant bacteria, which has become a global concern in production animals like swine [10, 11]. Consequently, the European legislation has been focused on regulating the use of antibiotics to minimize the development of antibiotic bacteria resistance [12]. Similarly, some countries have established national strategies to reduce the use of antibiotics in animal production [13–16]. In this context, it is essential to ascertain the extent of antibiotic resistance in order to effectively treat sick pigs, mitigate the impact of disease and prevent the development of antibiotic resistance.

Continuous surveillance is essential to evaluate the level of antibiotic resistance and to establish the most appropriate treatment for each swine farm, both in individual pigs as well as in disease outbreaks. Phenotypic screening using fecal swabs from individual pigs has traditionally constituted the primary method of surveillance [17]. Genotypic screening is much less common but provides epidemiological information from farms. In the case of Enterobacteriaceae, testing simultaneously phenotypic and genotypic resistance gains importance due to their role in the transmission of multidrug resistance genes, as they contaminate the environment in which pigs are kept and can spread these genes to the natural environment or even transfer them to other bacterial species [4, 17, 18].

Surveillance measures in swine farms are costly and labor-intensive as they require monitoring a significant number of pigs to obtain representative data [19, 20]. In order to minimize this limitation, oral fluids, tongues or air samples have been proposed and evaluated for some pig diseases [21–23]. Among them, air sampling has a high potential for surveillance as it may serve not only to detect pathogens involved in different disorders such as respiratory or digestive [24, 25], but also to assess the presence of antibiotic resistances. However, regarding pathogens involved in PWD, evaluating antibiotic resistance has only been applied to Enterobacteriaceae cultured from air samples [26–30]. This may compromise the sensitivity of the detection and underestimate the extent of antibiotic resistances as the process of air sampling can reduce the bacteria viability [31–33].

Against this background, the purpose of the present study was to evaluate the genotypic and phenotypic resistance of pathogenic Enterobacteriaceae isolated from piglets in weaning phase from the Northwest of Iberian Peninsula, as well as to explore the use of air as potential tool to estimate the Enterobacteriaceae antibiotic resistance levels on swine farms.

## Methods

### Sampling procedure

The study was conducted in seven farrow-to-wean farms located in the Northwest of the Iberian Peninsula. Five farms were affected by an outbreak of postweaning diarrhea (more than 15% of piglets with clinical diarrhea) or had implemented a control program against this disease, while the two remaining farms were included as control. Control farms were required to have not reported clinical outbreaks of PWD for at least one year. Table 1 shows a detailed description of the characteristics of the included farms.

**Table 1:** Characteristics of the farms

Farm	A	B	C	D	E	F	G
Sow census	130	100	550	470	700	200	450
N of piglets in weaning room	140	110	410	180	260	275	230
Farm management^±^	3-weeks batches	3-weeks batches	3-weeks batches	1-week batches	1-week batches	3-weeks batches	1-week batches
Age piglets sampled	6 weeks	6 weeks	6 weeks	6 weeks	6 weeks	6 weeks	7 weeks
Postweaning diarrhea situation	Outbreak	Outbreak	Outbreak	Piglets vaccinated*	Piglets vaccinated*	No clinical diarrhea	No clinical diarrhea
% aprox. piglets with diarrhea	25	20	40	10	5	0	0

*Vaccinated with Coliprotec F4/F18 at 21 days of age

^±^ It refers to the time interval between successive farrowing or weaning batches

We visited each farm once and took two types of samples: fecal swabs from piglets in the weaning phase (9 to 21 piglets), and two air samples from the room where these piglets were located. For the air samples we used the Coriolis µ Air Sampler (Bertin Instruments, Montigny-le‐Bretonneux, France). It was placed in the middle of the room at approximately 1 m height, with an air flow rate of 300 L per minute being used. The samples were collected in sterile cones (Coriolis Cones & Caps, Bertin Instruments, Montigny‐le‐Bretonneux, France) filled with 5 and 12 ml of sterile PBS, for a volume of air of 1.5 m and 9 m^3^, respectively. All swabs and cones were kept refrigerated and were submitted to the laboratory within the 24 h post-collection.

### Enterobacteriaceae isolation and pathogenic *E. coli* identification

Fecal swabs were plated onto MacConkey agar plates (VWR Chemicals, Leuven, Belgium) and incubated at 37 °C overnight. From each plate, one lactose-positive compatible colony was isolated and re-cultured onto MacConkey agar and identified biochemically with an API system (API 20E, bioMérieux, Marcy l’Etoile, France).

The Enterobacteriaceae confirmed as *E. coli* were subsequently screened by qPCR for the detection of the following virulence factors: STa, STb, LT, F4, F18 and Stx2. This was performed in order to identify pathogenic *E. coli* strains and exclude those that are commensal. DNA extraction was performed following the procolor recommended by Nakai et al. [34]. Briefly, a loopful of bacterial growth from each isolate was suspended in a 1.5 mL microtube containing 200 µL of Tris-EDTA buffer (Sigma-Aldrich, St. Louis, MO, USA). The tubes were incubated at 100 °C for 10 min and subsequently placed on ice for another 10 min. Finally, the lysates were centrifuged at 12.000 x g for five minutes and 100 µL of supernatant were transferred to a clean microtube and kept at − 80 °C until molecular analysis.

DNA samples were analyzed using the commercial qPCR kits: EXOone Sta + Stb + LT, EXOone F18 + Stx2 + EC and EXOone F4 (Exopol SL, Zaragoza, Spain), following the manufacturer’s instructions. qPCR positive and negative controls were supplied by the manufacturer and were used in each run. In addition, a specific primer for the *gadAB* gene of *E. coli* was used as an internal positive control. All reactions were run on an Applied Biosystems QuantStudio 5 (Thermo Fisher Scientific, Waltham, MA, USA).

### DNA extraction from air samples

The 14 cones with the air samples were vortexed and an elution of 1 ml of each one was transferred to a sterile Eppendorf tube and kept at − 80 °C until the DNA extraction was performed. For the DNA extraction we used the commercial extraction kit High Pure PCR Template Preparation Kit (Roche Diagnostics GmbH, Mannheim, Germany) following the manufacturer’s instructions indicated for tissue samples, using 200 µl of each air sample as starting material, and collecting the extracted DNA in 100 µl of elution buffer. The obtained DNA was kept at − 80 °C.

### *E. coli* susceptibility testing

*E. coli* strains in which at least one virulence factor was detected were cultured in sheep-blood agar. Two-three colonies from this culture were suspended in 2 ml of sterile saline solution to achieve a McFarland turbidity ranging from 0.5 to 1.0. Antimicrobial susceptibility testing was performed using the Kirby-Bauer disk diffusion method in Mueller Hinton agar, in accordance with the Clinical and Laboratory Standards Institute guidelines, Société Française de Microbiologie and European Committee on Antimicrobial Susceptibility Testing [35–37] (diameter breakpoints are indicated in Additional File 1). Table 2 shows the eight classes of antibiotics and the 24 antibiotics that were tested. After 24 h at 37 °C, the diameter of the inhibition zone around the disk was measured and the strain was classified as susceptible, resistant, or intermediate resistance to each antibiotic in accordance with the guidelines used. A strain was considered to have resistance to a class of antibiotics if it showed at least intermediate resistance to one of the antibiotics included in the group.

**Table 2:** Antibiotics and antibiotic resistance genes tested

Antibiotic class	Antibiotics (µg/disk)	Antibiotic resistance genes tested in E. coli strains	Antibiotic resistance genes tested in air samples
Penicillins	Ampicillin (10) Amoxicillin + clavulanic acid (20/10) Ticarcillin (75) Piperacillin (100) Piperacillin + tazobactam (100/10)	*bla*_TEM_ *bla*_SHV_ *bla*_NDM_ *ampC*	*bla*_TEM_ *bla*_SHV_ *ampC*
Cephalosporins	Cephalexin (30) Cefuroxime (30) Cefoxitin (30) Cefotaxime (30) Cefepime (30)	*ampC* *bla*_CTX−universal_ *bla*_CTX−M9_* *bla*_CTX−M15_*	*ampC* *bla*_CTX−universal_
Carbapenems	Imipenem (10)	-	-
Aminoglycosides	Gentamicin (10) Tobramycin (10) Kanamycin (30) Streptomycin (10) Neomycin (30) Apramycin (15)	*aac*-(3)-II *aac*-(3)-IV aac(6’)-aph(2”) *aac6’-Ib* *ant*2 *ant*6 *aph(6)-la* ^α^ *aph(6)-Id* ^*β*^	*aac*-(3)-II *aac*-(3)-IV
Quinolones	Nalidixic acid (30) Enrofloxacin (5) Marbofloxacin (5)	*qnrS* *parC* *qep*A	*qnrS* *parC*
Tetracyclines	Tetracycline (30) Doxycycline (30)	*tet*(A) *tet*(B) *tet*(M)	*tet*(A) *tet*(B)
Lipopeptides	Colistin (50)	*mcr*-1 *mcr*-2 *mcr*-4 *mcr*-5	*mcr*-1
Folate Pathway inhibitors	Trimethoprim- sulfamethoxazole (1.25/23.75)	*sul*1 *sul*2 *sul*3	*sul*1 *sul*2

*only tested in those *E. coli* strains positive to *bla*_CTX−Universal_

^α^ Previously designated as *strA;* ^*β*^ Previously designated as *str*B

### Characterization of antibiotic resistance genes

The presence of genes encoding resistance to antibiotics was tested by PCR. Regarding *E. coli* strains, the presence of genes associated with resistance to each group of antibiotics (Table 2) was tested if the strain showed total or intermediate phenotypic resistance to at least one antibiotic of the class. If the strain showed phenotypic susceptibility to all antibiotics of the class, it was considered that it did not encode any resistance gene. Regarding the air samples, only the genes indicated in Table 2 were tested.

Each PCR mixture contained 5 µL of PCR buffer, 1 µL of 2 mM of dNTP, 1 µL of each primer, 30.2 µL of sterile distilled water, 0.3 µL of Taq DNA polymerase, 1.5 µL of MgCl_2_ and 10 µL of DNA extracted from each *E. coli* strain or each air sample. Positive and negative controls of each gene from the Universidade de Trás-os-Montes e Alto Douro were used in each PCR run. Primer sequence and amplicon size for each primer are indicated in Table 3. PCR products were separated by electrophoresis on 1% agarose gels stained with RedSafe and visualized by ultraviolet light.

**Table 3:** Primers used to detect Enterobacteriaceae antibiotic genes

Target gene	Primers sequence (5’→3’)	Amplicon size (base pairs)	Reference
*bla*_TEM_	F: ATTCTTGAAGACGAAAGGGC R: ACGCTCAGTGGAACGAAAAC	1150	[38]
*bla*_SHV_	F: CACTCAAGGATGTATTGTG R: TTAGCGTTGCCAGTGCTCG	885	[38]
*bla*_NDM_	F: GGTTTGGCGATCTGGTTTTC R: CGGAATGGCTCATCACGATC	621	[39]
*ampC*	F: AATGGGTTTTCTACGGTCTG R: GGGCAGCAAATGTGGAGCAA	191	[40]
*bla*_CTX−universal_	F: CGATGTGCAGTACCAGTAA R: TTAGTGACCAGAATCAGCGG	585	[41]
*bla*_CTX−M9_	F: GTGACAAAGAGAGTGCAACGG R: ATGATTCTCGCCGCTGAAGCC	857	[42]
*bla*_CTX−M15_*	F: CACACGTGGAATTTAGGGACT R: GCCGTCTAAGGCGATAAACA	995	[43]
*aac*-(3)-II	F: ACTGTGATGGGATACGCGTC R: CTCCGTCAGCGTTTCAGCTA	237	[38]
*aac*-(3)-IV	F: CTTCAGGATGGCAAGTTGGT R: TCATCTCGTTCTCCGCTCAT	286	[38]
*aac(6’)-aph(2”)*	F: CCAAGAGCAATAAGGGCATA R: CACTATCATAACCACTACCG	220	[44]
*aac6’-Ib*	F: TTGCGATGCTCTATGAGTGGC R: TACTCGAATGCCTGGCGTGTTT	482	[45]
*ant*2	F: ATGTTACGCAGCAGGGCAGTCG R: CGTCAGATCAATATCATCGTGC	188	[46]
*ant*6	F: ACTGGCTTAATCAATTTGGG R: GCCTTTCCGCCACCTCACCG	577	[47]
aph(6)-la *	F: ATTCTGACTGGTTGCCTGTC R: CGCAGATAGAAGGCAAGG	815	[38]
*aph(6)-Id ±*	F: TTCTCATTGCGGACAACCT R: TAGATCGCGTTGCTCCTCTT	747	[38]
*qnrS*	F: GCAAGTTCATTGAACAGGGT R: TCTAAACCGTCGAGTTCGGCG	428	[48]
*parC*	F: AAACCTGTTCAGCGCCGCATT R: GTGGTGCCGTTAAGCAAA	395	[49]
*qep*A	F: GCAGGTCCAGCAGCGGGTAG R: GGACATCTACGGCTTCTTCG	617	[50]
*tet*(A)	F: GTAATTCTGAGCACTGTCGC R: CTGTCCTGGACAACATTGCTT	937	[38]
*tet*(B)	F: CTCAGTATTCCAAGCCTTTG R: CTAAGCACTTGTCTCCTGTT	416	[38]
*tet*(M)	F: GTTAAATAGTGTTCTTGGAG R: CTAAGATATGGCTCTAACAA	576	[51]
*mcr*-1	F: AGTCCGTTTGTTCTTGTGGC R: AGATCCTTGGTCTCGGCTTG	320	[52]
*mcr*-2	F: CGACCAAGCCGAGTCTAAGG R: CAACTGCGACCAACACACTT	92	[53]
*mcr*-4	F: AGAATGCCAGTCGTAACCCG R: GCGAGGATCATAGTCTGCCC	230	[53]
*mcr*-5	F: ATGCGGTTGTCTGCATTTATC R: TCATTGTGGTTGTCCTTTTCTG	1644	[54]
*sul*1	F: TGGTGACGGTGTTCGGCATTC R: GCGAGGGTTTCCGAGAAGGTG	789	[55]
*sul*2	F: CGGCATCGTCAACATAACC R: GTGTGCGGATGAAGTCAG	722	[38]
*sul*3	F: CATTCTAGAAAACAGTCGTAGTTCG R: ATCTGCAGCTAACCTAGGGCTTTGGA	990	[38]

* Previously designated as *strA; ±* Previously designated as *str*B

### Statistical analysis

A logistic regression was used to evaluate the presence of differences in the proportion of pathogenic *E. coli* strains with phenotypic and genotypic resistance between antibiotic class. Regarding phenotypic resistance, those strains with intermediate resistance according to the guidelines were considered as resistant ones.

We explored the presence of clustering in the phenotypic and genotypic resistance by farm by using heat maps. Cluster analysis was made using the complete linkage method using the the R package gplots for this analysis [56]. Furthermore, we studied the co-occurrence of resistance genes to determine whether a particular pair of genes significantly occurred together (positive association), or at random, by testing whether the observed frequency of pairwise co-occurrence is greater or lesser than expected given the overall prevalence of pathogen at an alpha threshold of 0.05 [57]. The co-occurrence analysis was performed using the “cooccur” package [58]. The frequency of pairwise co-occurrences were also used to build a network, which was represented using the program Gephi [59].

The concordance between the results of pathogenic *E. coli* and the air samples was performed at farm level considering the antibiotic class. A farm was considered phenotypically resistant to a class of antibiotics if at least one strain showed phenotypic resistance to an antibiotic of that class. Similarly, a farm was considered genotypically resistant to a class of antibiotics if at least one strain encoded a resistance gene to that antibiotic class. Regarding air sampling, a farm was considered resistant to a class of antibiotic if the air sample was positive to at least one antibiotic resistance gene of the corresponding class. Thus, to evaluate this concordance, the kappa coefficient was used [60].

All data analysis were performed in R v. 4.1.1 [61] (Vienna, Austria). In all cases, a *p* < 0.05 was considered as significant.

## Results

### Enterobacteriaceae isolation

A total of 111 fecal swabs were processed. The 111 Enterobacteriaceae isolated were classified as *E. coli*. Of those *E. coli* strains, 43 (38,74% of the total) were identified as pathogenic *E. coli* (range 2 to 11 strains per farm). In most farms we observed different profiles of virulence factors, as well as between farms: Farm A (STb, Lt, F18), Farm B (STb; STb, Lt, F18), Farm C (STa, STb, F4), Farm D (F18; STb; STa, STb; STb, Lt, F18; STa, STb, F18), Farm E (STb; STa, STb, F4), Farm F (STb; STb, Lt, F18), Farm G (STb; STa, STb, F18).

### Phenotypic antibiotic resistance of pathogenic *E. coli*

**Fig. 1:**
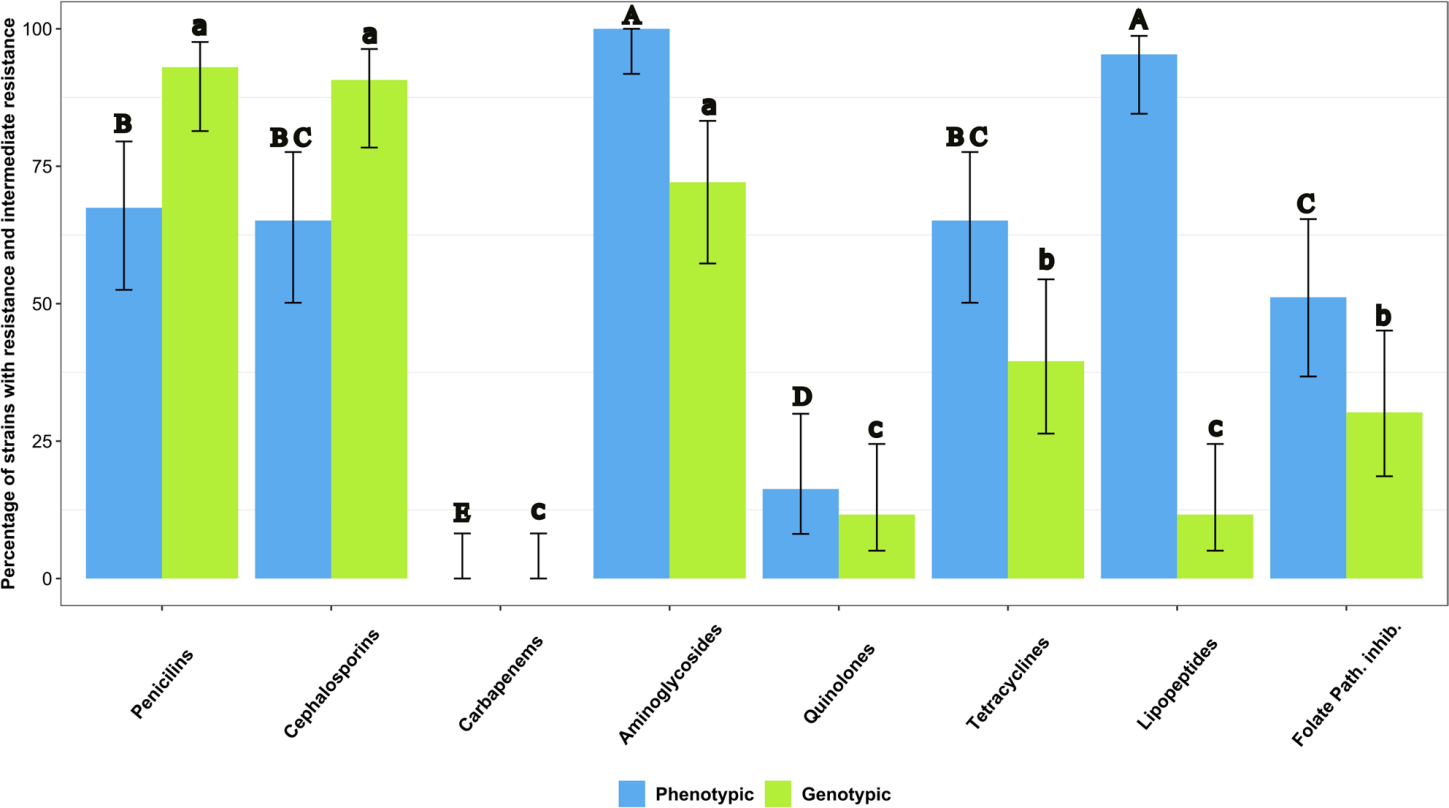
Percentage of pathogenic *E. coli* strains with phenotypic and genotypic resistance to each antibiotic class. Different letters indicate significant differences between antibiotic class (Capital letters: phenotypic resistance; lowercase letters: genotypic resistance). Strains with intermediate phenotypic resistance were considered resistant strains

The 43 pathogenic *E. coli* strains showed phenotypic resistance at total or intermediate level to at least 2 of the 8 classes of antibiotics tested, being aminoglycosides and lipopeptides the antibiotic classes with the higher percentage of resistant strains (*p* < 0.05). On the contrary, carbapenems were the only antibiotic class to which resistance was not detected (Fig. 1).

**Fig. 2:**
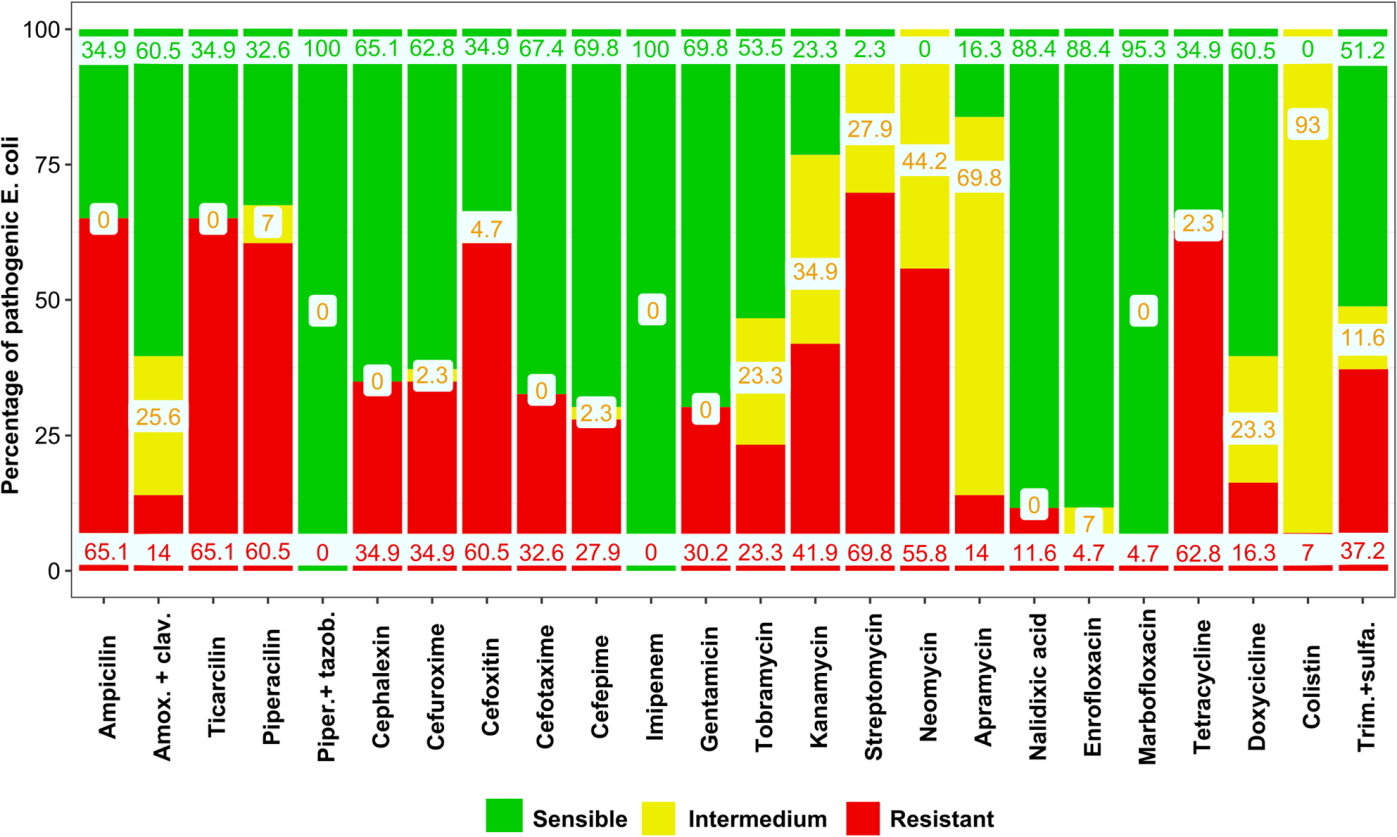
Percentage of pathogenic *E. coli* strains susceptible, intermediate resistance or resistant to each antibiotic

Regarding individual antibiotics, streptomycin (69.8%), ampicillin (65.1%), ticarcillin (65.1%), tetracycline (62.8%), piperacillin (60.5%), cefoxitin (60.5%) and neomycin (55.8%) were the antibiotics to which *E. coli* showed a higher frequency of resistance. Piperacillin with tazobactam (0.0%) imipenem (0.0%), marbofloxacin (4.7%), enrofloxacin (4.7%) and colistin (6.98%) were those to which we observed a lower frequency of resistance. In the rest of tested antibiotics, the frequency of resistance ranged between 11.6% against nalidixic acid to 41.9% against kanamycin (Fig. 2). If considering both the intermediate resistance, colistin, neomycin, streptomycin, apramycin and kanamycin showed the higher values (100%, 97.8%, 83.8% and 76.8%, respectively (Fig. 2).

**Fig. 3:**
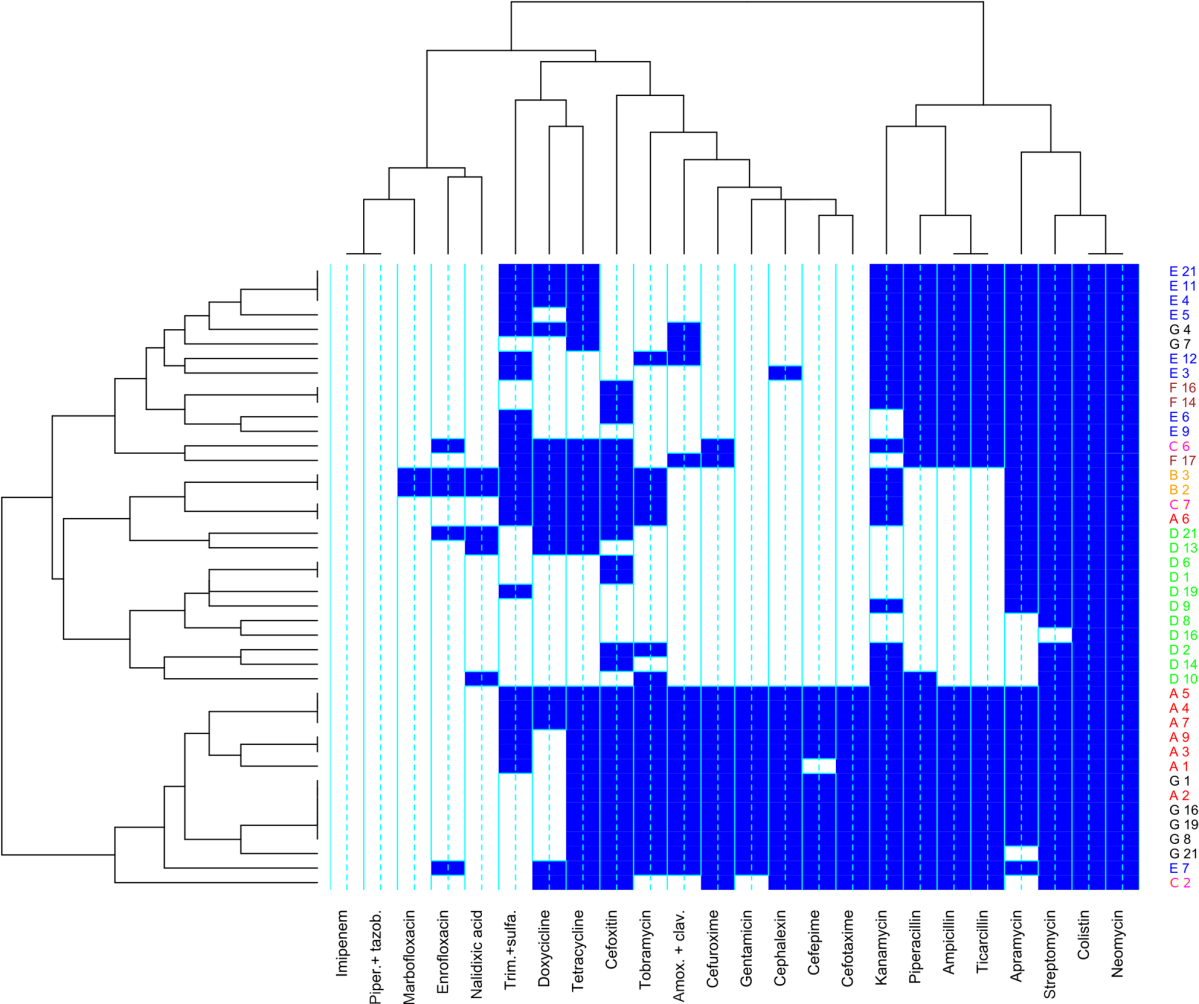
Heat map of pathogenic *E. coli* isolated from fecal samples, showing clustering by farm and by phenotypic resistance. Letters indicate the farm from which isolate was obtained and different colors indicate strains from different farms. Strains with intermediate resistance were considered resistant. Blue or white box indicate resistance or susceptibility, respectively

When we classified the isolates according to their phenotypic resistance profile, we observed that pathogenic *E. coli* tended to cluster according to their farm of origin (Fig. 3). However, in most farms isolated strains were detected that showed a different profile, standing out from the others. When considering the isolates phenotypic resistance profile, two main clades with two subclades were described regarding the phenotypic resistance: the most frequent combination was presenting resistance against neomycin, colistin, streptomycin and apramycin, following by another subclade with resistance to most of penicillins and kanamycin. In contrast, resistance to imipenem, piperacillin with tazobactam, and quinolones was located at the opposite side of the heatmap, representing a completely different pattern from that observed in the first two clades. Regarding tetracycline phenotypic resistance, despite being one of the most frequent, it was clustered nearest the cephalosporin class and near the quinolones than the majority of penicillins and aminoglycosides. Additional File 2 provides a detailed profile of phenotypic antibiotic resistance detected on each farm. Finally, no clustering was observed due to farm health situation (diarrhea outbreak, vaccinated or control).

### Genotypic antibiotic resistance of pathogenic *E. coli*

**Fig. 4:**
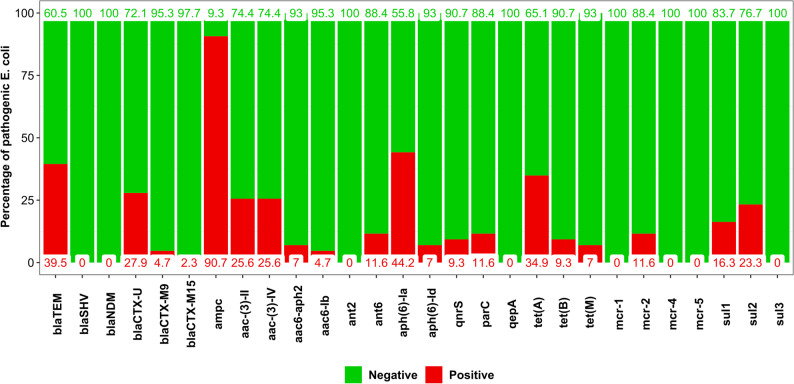
Percentage of pathogenic *E. coli* strains that encodes each antibiotic resistance gene

The genes that encode resistance against penicillins, cephalosporins and aminoglycosides were the most detected (*p* < 0.05), while those genes that encode resistance against lipopeptides, quinolones and carbapenems were the least frequent (*p* < 0.05) (Fig. 1). The presence of genes against carbapenems was not evaluated because all strains were phenotypically susceptible to imipenem. From the 43 pathogenic *E. coli* strains, 42 (97.7%) were positive to at least one antibiotic resistance gene, and the most common was *ampC*, present in 90.7% of the pathogenic strains. The genes *aph(6*)*-la*, *bla*_TEM_ and *tet*(A) were the following more common, present in the 44.2%, 39.5% and 34.9% of the strains, respectively. Instead, the genes *bla*_SHV_, *bla*_NDM_, *ant*2, *qep*A, *mcr-*1, *mcr-*4, *mcr-*5 and *sul*3 were not detected in any strain (Fig. 4).

**Fig. 5:**
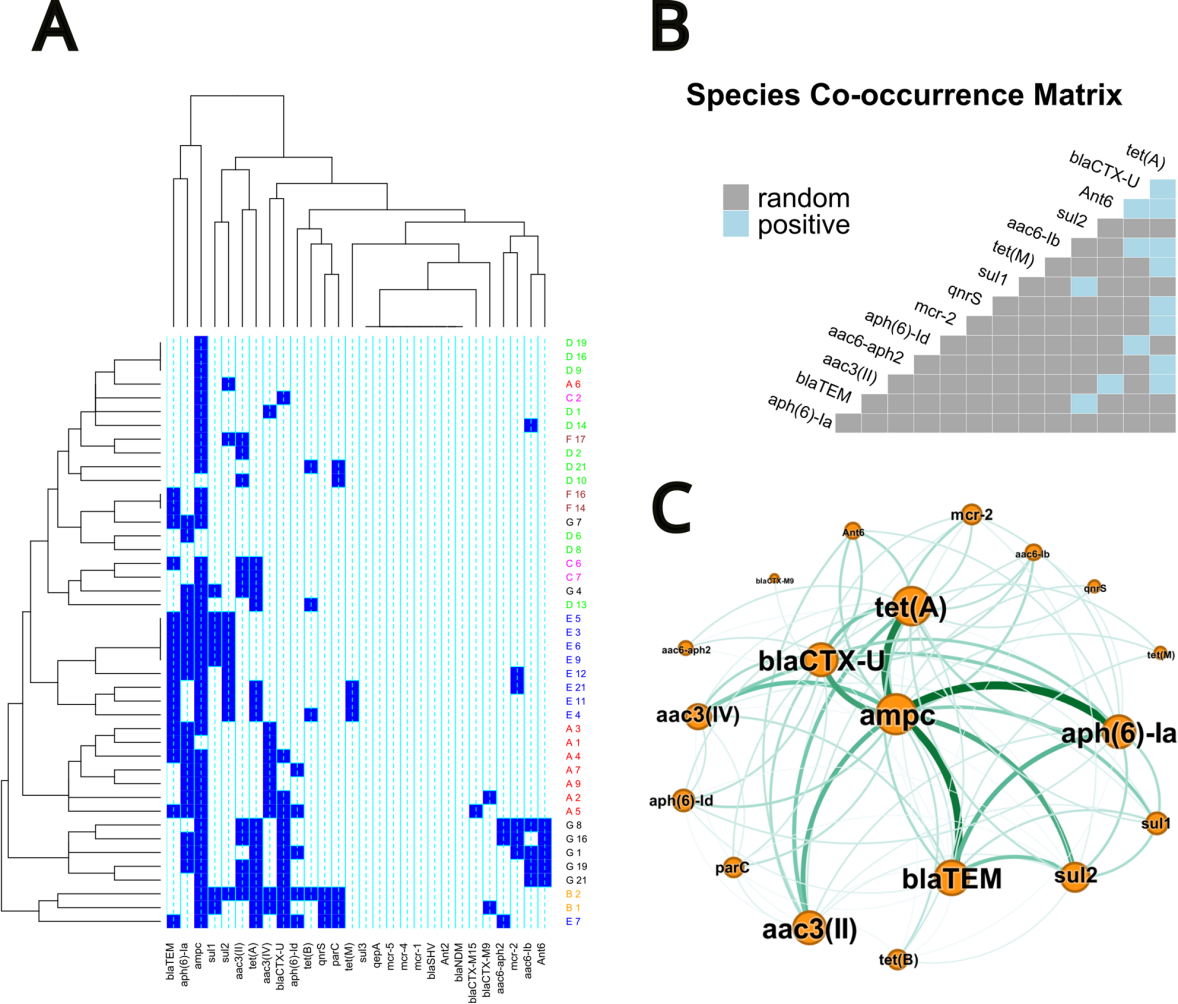
Epidemiological description of *E. coli* resistance genes. (**A**) Heat map with clusters according to farms and genes frequency. Blue or white box indicate a positive or negative result, respectively. Different colors indicate strains from different farms. (**B**) Matrix of co-occurrence. Blue color indicates significant results (*p* < 0.05). (**C**) Netplot of antibiotic resistance genes co-occurrence. Larger circles indicate a higher frequency of the gene. The intensity of the lines represents the frequency of the co-occurrence: *ampC* with *aph* *(6)-la*, *bla*_TEM_, *tet*(A), *bla*_CTX−universal_, *sul*2, *aac-* *(3)*-II, *aac-* *(3)*-IV, and *bla*_TEM_ with *aph* *(6)-la* were the most common

The heatmap revealed a strong clustering of pathogenic *E. coli* strains according to their farm of origin (Fig. 5A). Nevertheless, with the exception of Farm B, strains showing different resistance gene profiles were also detected in the other farms and were classified into subclades distinct from the main clusters. In addition, the postweaning diarrhea status in the farm did not affect to the clusters of strains as no relationship were observed between farms with the same situation (outbreak, vaccinated or control). Of the 43 strains, 22 (51.16%) encode resistance genes against four of the antibiotic classes tested. Figure 5.A also shows a main cluster of antibiotic resistance genes, which includes *bla*_TEM_, *aph(6)* *-la* and *ampC*, while the remaining genes are classified in a heterogeneous cluster. Despite this heterogeneity, significant associations were detected between pairwise genes (*p* < 0.05), being *tet*(A) and *bla*_CTX−Universal_ the genes most frequently associated with others (Fig. 5. B and C). Supplementary Material 2 provides a detailed profile of genotypic antibiotic resistance detected on each farm.

### Detection of antibiotic resistance genes of pathogenic *E. coli* in the air at farm level

Of the 13 genes evaluated in air samples, 11 were present in pathogenic *E. coli* from at least two out of the seven studied farms. Regarding the air samples, similar detection rates were obtained when sampling 1.5 or 9 m^3^ of air for all genes, although in a few cases the 9 m^3^ air sample allowed the detection of a slightly higher number of farms positive to each gene (Table 4). In addition, it was observed a different detection pattern in the air sampling depending on the gene: while *ampC*, *aac*-(3)-IV and *sul*2 were detected in the air from all farms in which they had been detected in pathogenic *E. coli*, other genes as *bla*_CTX−universal_, *tet*(A) and *tet*(B) were not detected in any air sample despite they were present in pathogenic *E. coli* from a considerable number of farms (Table 4). Additionally, other genes such as *aac*-(3)-IV, *qnrS*, *par*C and also *sul*1 were detected in the air of some farms in which they were not detected in pathogenic *E. coli*.

The concordance between phenotypic and genotypic resistance in pathogenic *E. coli* and the airborne genes detected was weak (k < 0.4 in all cases). Specifically, regarding phenotypic resistance the obtained values were k = 0.04 and k = 0.167 for 1.5 and 9 m^3^ of air, respectively; regarding genotypic resistance the values were 0.138 and 0.339 for 1.5 and 9 m^3^ of air, respectively, with *p* < 0.05 in the last case.

**Table 4:** Number of farms in which each gene was detected in pathogenic *E. coli* strains and in air samples (1.5 and 9 m³)

Gene	E. coli	1.5 m^3^ of air			9 m^3^ of air		
	*n* farms gene + / *n* total farms	*n* farms air + / *n* total farms	*n* farms air + / *n* farms with E. coli +	*n* farms air + / *n* farms with E. coli -	*n* farms air + / *n* total farms	*n* farms air + / *n* farms with E. coli +	*n* farms air + / *n* farms with E. coli -
*bla*_TEM_	5 / 7	1 / 7	1 / 5	0 / 2	1 / 7	1 / 5	0 / 2
*bla*_SHV_	0 / 7	0 / 7	0 / 0	0 / 7	0 / 7	0 / 0	0 / 7
*ampC*	7 / 7	4 / 7	4 / 7	0 / 0	7 / 7	7 / 7	0 / 0
*bla*_CTX−universal_	5 / 7	0 / 7	0 / 5	0 / 2	0 / 7	0 / 5	0 / 2
*aac*-(3)-II	5 / 7	5 / 7	4 / 5	1 / 2	4 / 7	4 / 5	0 / 2
*aac*-(3)-IV	3 / 7	5 / 7	2 / 3	3 / 4	5 / 7	3 / 3	2 / 4
*qnrS*	2 / 7	4 / 7	1 / 2	3 / 5	4 / 7	1 / 2	3 / 5
*parC*	3 / 7	3 / 7	1 / 3	2 / 4	4 / 7	2 / 3	2 / 4
*tet*(A)	5 / 7	0 / 7	0 / 5	0 / 2	0 / 7	0 / 5	0 / 2
*tet*(B)	3 / 7	0 / 7	0 / 3	0 / 4	0 / 7	0 / 3	0 / 4
*mcr*-1	0 / 7	0 / 7	0 / 0	0 / 7	0 / 7	0 / 0	0 / 7
*sul*1	3 / 7	2 / 7	1 / 3	1 / 4	3 / 7	1 / 3	2 / 4
*sul*2	4 / 7	6 / 7	4 / 4	2 / 3	6 / 7	4 / 4	2 / 3

For air samples, the results are presented separately for farms with at least one pathogenic *E. coli* strain positive for each gene, and for farms where all strains were negative

## Discussion

In the present study *E. coli* was the predominant coliform bacteria from fecal swabs under the selective culture conditions employed. The presence of pathogenic *E. coli* in both diarrheic and healthy piglets is well-documented, highlighting its role as primary agent in PWD outbreaks and as an endemic pathogen in swine farms [62, 63]. We detected a considerable variety of pathogenic strains with different virulence factors, consistent with previous findings [64]. However, despite the variety of virulence factors, nearly all the strains of the same farm were clustered together based on their phenotype and genotype antibiotic resistance patterns. It has been described that the use of specific antibiotics over time leads to the development of different resistance profiles shaped by each farm’s history of antibiotic use [65]. Thus, given these factors, the lack of clustering according to the farm health status (outbreak, vaccination and control) was not surprising.

Our results indicate that pathogenic strains of *E. coli* show a remarkable phenotypic and genotypic resistance across multiple classes of antibiotics. Notably, all strains in the present study showed complete or intermediate phenotypic resistance to lipopeptides and aminoglycosides, and the majority also, at a lower level, to other classes of antibiotics. The high level of resistance to lipopeptides is particularly striking, especially considering that since 2016 the use of colistin in swine production in Spain has been significantly reduced (a decrease of 97.18% from 2015 to 2018), and it is likely that this reduction has continued to the present year [66]. So, we cannot rule out that the guideline used for colistin in the susceptibility testing [36] may have influenced our results. We used the disk diffusion method to evaluate phenotypic resistance to colistin instead the gold standard broth microdilution method. It is known that colistin has pharmacodynamic characteristics that limit its diffusion in agar, making the standardization of susceptibility guidelines challenging [67]. Importantly, phenotypic resistance to colistin may be driven by mechanisms other than *mcr* genes, and phenotypic results are generally considered more reliable than genotypic data. Thus, although they showed high phenotypic resistance, only 11.2% of the pathogenic E. coli strains encoded resistance genes to lipopeptides (specifically the *mcr*-2 gene), which is one of the antibiotic classes with the lowest genotypic resistance. Nonetheless, it is remarkable that despite in the last decade the sales of colistin are almost nil in swine farms [68], the *mcr*-2 gene is detected in year 2024 in pathogenic *E. coli* strains isolated from weaned piglets. This gene is rarely described in *E. coli* isolated from pigs in Europe [69]. In addition, it should not be overlooked that the situation regarding colistin resistance genes in the present study is markedly different to that reported by Aguirre et al. [70]: in that study the authors detected *mcr* genes in a higher percentage of *E. coli* strains than ours (21.5% vs. 11.2%), and they detected *mcr*-1, *mcr*-4 and *mcr*-5 genes but not the *mcr*-2 gene. It cannot be ruled out that, although the farms included in both studies were located in Iberian Peninsula, variables such as the different pig farm density and animal movements, herd census and, above all, differences in antibiotic treatment practices may explain the discrepancy observed. This discrepancy highlights the importance of continuous surveillance of antibiotic resistance genes in swine farms, especially for those antibiotics considered critically important for human medicine.

Regarding the aminoglycosides, our study shows a high percentage of phenotypic and genotypic resistance in pathogenic *E. coli*. In addition, the high variability of resistance genes is notable, as 7 out of 8 tested genes were detected. This is consistent with the knowledge on the multiple resistance mechanisms against aminoglycosides [71, 72]. This also suggests that various resistance mechanisms may be circulating in pathogenic *E. coli* in each swine farm, jeopardizing the efficacy of this antibiotic class. This hypothesis is further supported by two other Spanish studies that reported substantial variability of aminoglycoside resistance genes in *E. coli* strains from pigs [73, 74]. However, in contrast to these studies, the most predominant gene in our study was *aph* *(6)-Ia*, whereas they highlighted *aph* *(6)-Id* among the genes analyzed in common. Considering this observation and the variability in aminoglycoside resistance genes, further research is needed in this field.

Penicillins, cephalosporins, tetracyclines and folate pathway inhibitors are antibiotics widely used in swine production and, considering recent studies, it was expected a considerable resistance in pathogenic *E. coli* [70, 75–79]. However, the level of resistance was, in general, lower than the observed aminoglycosides. Regarding genotypic resistance, the highest value was observed both in penicillins and cephalosporins. This observation is influenced by the gene *ampC*, which encodes resistance for both families [80]. This gene was detected in more than 90% of pathogenic *E. coli* strains. This remarkable prevalence is consistent with the values reported in other studies [75, 79]. The widespread presence of *ampC* gene, particularly when co-occurring with *bla*_CTX−universal_ and/or *bla*_TEM_ seems to be the main factor underlying the considerable proportion of strains resistant to third generation onwards cephalosporins, as well as piperacillin and ticarcillin, antibiotics whose resistance has significant public health implications [81]. However, the ubiquity of *ampC* limits its relevance in co-occurrence analysis, as it is present in nearly all strains. Among the genes analyzed, *tet*(A) is notable, showing a positive association with genes conferring resistance to multiple antibiotic classes. This suggests that the presence of *tet*(A) may serve as indicator of reduced susceptibility to other antibiotic classes as well.

The present paper also shows an absence of resistance against piperacillin with tazobactam and imipenem, as well as negative results against the gene *bla*_NDM_, in pathogenic *E. coli*. Similarly, we also observed relatively low resistance against all quinolones. These are noteworthy results as they are indicative that in included swine farms from the Northwestern Iberian Peninsula the level of resistance against critical antibiotics in human health is minimal. In fact, piperacillin with tazobactam and imipenem are not authorized for veterinary use, but were included in the susceptibility test to evaluate the presence of advance resistance mechanisms against beta-lactam antibiotics and carbapenems [82]. In regards to carbapenems, curiously our results are consistent with other studies performed in Spanish swine farms, but different to the observed in farms from central Portugal [75, 76, 83]: a complete absence of resistance to this antibiotic class in Spanish swine farms but a considerable level of resistance in central Portugal. Therefore, we cannot discard that this notable difference between neighbor countries could be related with the absence of national guideline for prudent antibiotic use in Portugal, unlike Spain [13, 84, 85]. A study including more herds as well as focusing on different antibiotic prescriptions in swine farms would be of interest in the future.

In regard to the results obtained from the farm air analysis, it should be noted that the methodology described requires further refinement before it can be considered a reliable alternative for evaluating the level of antibiotic resistance in pathogenic *E. coli*. Unlike analysis of fecal swab cultures, the results of qPCR in air samples reflect the presence of both viable and non-viable bacteria. It must be remarked that only genes specific or highly associated with Enterobacteriaceae family were tested in air samples, approach that was intentionally chosen to avoid potential interference from Gram-positive bacteria, which are predominant in airborne microbiota from pig housing environment. Thus, the best concordance was obtained when sampling 9 m^3^ of air and using the farm genotypic resistance as reference but, even in this situation, the obtained kappa was low. However, this low concordance seems to be influenced by the different performance of some antibiotic genes in air samples regarding its detection in *E. coli* strains. It is highly remarkable that genes such as *tet*(A), *tet*(B) and *bla*_CTX−universal_ were not detected in any air sample, and *bla*_TEM_ was detected in only in one farm. Instead, the 9 m^3^ air sample was positive in all farms for *ampC*, *aac*-3-(IV) and *sul*2. All the mentioned genes were present in most farms and in a considerable proportion of pathogenic *E. coli* strains. This observation is similar to the described by Gilbert et al. (2010), who also did not detect genes such as *tet*(A) in air samples, despite being present in bacteria strains from biofilm, but differs from other studies which detect *tet*(B) [86, 87]. A higher proportion of bacteria that encodes *tet*(B) in that studies may be behind this discrepancy. Therefore, our study suggests that air sampling could have a better performance to detect some Enterobacteriaceae resistance genes than others.

This different performance of antibiotic resistance genes in air samples may be influenced by several factors, such as the low amount of bacterial DNA present in airborne particles, potential losses during DNA extraction, the presence of PCR inhibitors, or the adsorption of DNA to dust particles, which could reduce its availability for amplification. Although we initially considered that plasmid-borne genes might be more exposed to environmental stress than chromosomal genes, and therefore more prone to degradation [88–90], we acknowledge that qPCR is able to detect DNA from viable and non-viable bacteria, as well as from plasmids, as long as it remains sufficiently intact. Thus, DNA degradation alone is unlikely to fully explain the differences observed between chromosomal and plasmid-associated genes.

The detection of resistance genes in air samples, despite not finding them in the isolated pathogenic *E. coli* may be explained by the fact of some genes examined in this study can also be encoded by commensal Enterobacteriaceae [10]. Thus, the performance of swine farm air as surveillance tool to characterize *E. coli* genes seems to be more conditioned in regard to viral infections. The detection of viral DNA or RNA is indicative of an infection on the farm, but in the detection of antibiotic resistance genes, it must be considered that they may come from pathogenic and/or commensal bacteria. Consequently, the use of air from pig farms to assess the level of resistance in pathogenic *E. coli* needs a refinement to become a viable alternative surveillance tool, thus representing a promising line of research for the future.

Finally, some aspects concerning the performance of the study must be mentioned. As previously mentioned, air and swab samples were not processed in the same way. While qPCR was performed directly on air samples, fecal swabs were cultured only after bacterial growth was analyzed. Consequently, the air analysis included both viable and non-viable microorganisms, whereas the culture based approach exclusively viable bacteria. For future studies, an interesting step forward would be to evaluate the detection of viable Enterobacteriaceae directly from air samples and to attempt both phenotypic and genotypic susceptibility testing from those isolates. Similarly, the analysis of additional environmental samples, such as swabs from soil or stable surfaces, could provide alternative insights. Finally, to better assess the potential of air and environmental samples as monitoring tools for antibiotic resistance, future research should consider genetic or genomic approaches on air-derived bacteria. For example, whole-genome sequencing or targeted resistome/metagenomic sequencing could help identify resistance genes and link airborne bacteria to farm-specific resistance profiles.

## Conclusions

Our findings demonstrate a high prevalence of antibiotic resistance in pathogenic *E. coli* isolated from weaning piglets, with aminoglycosides being of particular concern, and this pattern was observed regardless of the presence of postweaning digestive disorders. Moreover, the study reveals a remarkable co-occurrence of major resistance genes against multiple antibiotic classes in a substantial proportion of pathogenic *E. coli* across most farms. These results underscore the urgent need for responsible antibiotic use policies and continuous resistance monitoring in swine production systems.

Regarding air sampling, the results indicate that this approach still requires further refinement before it can serve as a reliable surveillance tool for antimicrobial resistance in pathogenic *E. coli*. Nonetheless, its performance varied depending on the resistance gene, with *ampC*,* aac-3*-(IV), and *sul*2 emerging as the most promising candidates, suggesting that air-based monitoring could become a valuable complementary strategy once methodological limitations are addressed.

## Supplementary Information

Below is the link to the electronic supplementary material.

Supplementary Material 1

Supplementary Material 2

## Data Availability

The data of this study are available from the corresponding author upon reasonable request.

## Declarations

### Generative AI and AI-assisted technologies in the writing process

The authors declare that they did not use generative artificial intelligence (AI) and AI-assisted technologies in any part of the study development and in the paper redaction.
