# Supplementary material for: Epidemiological trends of antibiotic resistance in pathogenic Escherichia coli in swine farms from the Northwest Iberian Peninsula and evaluation of air sampling for antibiotic resistance surveillance
Source: Porcine Health Manag. 2025 Dec 29;11:64. doi: 10.1186/s40813-025-00475-0 (PMC12751166; doi:10.1186/s40813-025-00475-0)
Supplement: Supplementary file 1 — Supplementary Material 1 [file 40813_2025_475_MOESM1_ESM.docx]

Additonal File 1. Guidelines for Enterobacteria Susceptibility Testing

| **Antibiotic Group** | **Antibiotic** | Disk content  (µg/disk) | **Zone diameter breakpoint (milimeter)** | | | Guideline |
| --- | --- | --- | --- | --- | --- | --- |
|  |  |  | **S (≥)** | **I** | **R (≤)** |  |
| **Penicilins** | **Ampicillin** | 10 | **17** | **14-16** | **13** | CLSI (2020) |
|  | **Amoxicillin + clavulanic acid** | 20/10 | **18** | **14-17** | **13** | CLSI (2020) |
|  | **Ticarcillin** | 75 | **23** | **21-22** | **20** | EUCAST (2022) |
|  | **Piperacillin** | 100 | **21** | **18-20** | **17** | CLSI (2020) |
|  | **Piperacillin + tazobactam** | 100/10 | **21** | **18-20** | **17** | CLSI (2020) |
| **Cephalosporins** | **Cephalexin** | 30 | **14** |  | **13** | EUCAST (2022) |
|  | **Cefuroxime** | 30 | **18** | **15-17** | **14** | CLSI (2020) |
|  | **Cefoxitin** | 30 | **18** | **15-17** | **14** | CLSI (2020) |
|  | **Cefotaxime** | 30 | **26** | **23-25** | **22** | CLSI (2020) |
|  | **Cefepime** | 30 | **25** | **19-24** | **18** | CLSI (2020) |
| **Carbapenems** | **Imipenem** | 10 | **23** | **20-22** | **19** | CLSI (2020) |
| **Aminoglycosides** | **Gentamicin** | 10 | **15** | **13-14** | **12** | CLSI (2020) |
|  | **Tobramycin** | 10 | **15** | **13-14** | **12** | CLSI (2020) |
|  | **Kanamycin** | 30 | **18** | **14-17** | **13** | CLSI (2020) |
|  | **Streptomycin** | 10 | **15** | **12-14** | **11** | CLSI (2020) |
|  | **Neomycin** | 30 | **17** | **15-16** | **14** | SFM (2021) |
|  | **Apramycin** | 15 | **15** | **12-14** | **11** | SFM (2021) |
| **Quinolones** | **Nalidixic Acid** | 30 | **19** | **14-18** | **13** | CLSI (2020) |
|  | **Enrofloxacin** | 5 | **23** | **17-22** | **16** | CLSI (2020) |
|  | **Marbofloxacin** | 5 | **20** | **15-19** | **14** | CLSI (2020) |
| **Tetracyclines** | **Tetracycline** | 30 | **15** | **12-14** | **11** | CLSI (2020) |
|  | **Doxycycline** | 30 | **14** | **11-13** | **10** | CLSI (2020) |
| **Lipopeptides** | **Colistin** | 50 | **18** | **15-17** | **14** | SFM (2021) |
| **Folate Pathway inhibitors** | **Trimethoprim- sulfamethoxazole** | 1.25/23.75 | **16** | **11-15** | **10** | CLSI (2020) |
