# Supplementary material for: Epidemiological trends of antibiotic resistance in pathogenic Escherichia coli in swine farms from the Northwest Iberian Peninsula and evaluation of air sampling for antibiotic resistance surveillance
Source: Porcine Health Manag. 2025 Dec 29;11:64. doi: 10.1186/s40813-025-00475-0 (PMC12751166; doi:10.1186/s40813-025-00475-0)
Supplement: Supplementary file 2 — Supplementary Material 2 [file 40813_2025_475_MOESM2_ESM.docx]

Additional File 2. Virulence factors, phenotypes and genotypes of *E. coli* detected on each farm

| Farm | Strain | Virulence factors | Phenotype (includes only resistant antibiotics according to guidelines) | Genotype |
| --- | --- | --- | --- | --- |
| A | A 1 | STb – LT – F18 | AMP – TIC – PIP – CFX – CXM – FOX – CTX – CN – TOB – K – S – NE – TE | *bla*_TEM_ – *aac*-(3)-IV – *aph(6)-la* |
|  | A 2 | STb – LT – F18 | AMP – TIC – PIP – CFX – CXM – FOX – CTX – CN – TOB – K – S – NE – APR – TE | *bla*_CTX-universal_ – *bla*_CTX-M9_ – *ampC* – *aac*-(3)-IV – *aph(6)-la* |
|  | A 3 | STb – LT – F18 | AMP – TIC – PIP – CFX – CXM – FOX – CTX – CPM – CN – TOB – K – S – NE – APR – TE | *bla*_TEM_ – *ampC* – *aac*-(3)-IV – *aph(6)-la* |
|  | A 4 | STb – LT – F18 | AMP – TIC – PIP – CFX – CXM – FOX – CTX – CPM – CN – TOB – K – S – NE – APR – TE | *bla*_TEM_ – *bla*_CTX-universal_ – *ampC* – *aac*-(3)-IV – *aph(6)-la* |
|  | A 5 | STb – LT – F18 | AMP – TIC – PIP – CFX – CXM – FOX – CTX – CPM – CN – TOB – K – S – NE – APR – TE | *bla*_TEM_ – *bla*_CTX-universal_ – *bla*_CTX-M15_ – *ampC* – *aac*-(3)-IV – *aph(6)-la* – *aph(6)-Id* |
|  | A 6 | STb – LT – F18 | FOX – S – TE – DO – SXT | *ampC* – *sul*2 |
|  | A 7 | STb – LT – F18 | AMP – TIC – PIP – CFX – CXM – FOX – CTX – CPM – CN – TOB – K – S – NE – APR – TE | *ampC* – *aac*-(3)-IV – *aph(6)-la* – *aph(6)-Id* |
|  | A 9 | STb – LT – F18 | AMP – TIC – PIP – CFX – CXM – FOX – CTX – CPM – CN – TOB – K – S – NE – APR – TE | *ampC* – *aac*-(3)-IV – *aph(6)-la* |
| B | B 1 | STb | FOX – TOB – K – S – NE – NA – ENR – MAR – TE – DO – SXT | *bla*_CTX-universal_ – *bla*_CTX-M9_ – *ampC* – *aac*-(3)-IV – *aph(6)-la* – *qnrS – parC –* *tet*(A) – *sul1* |
|  | B 2 | STb – LT – F18 | FOX – TOB – K – S – NE – NA – ENR – MAR – TE – DO – SXT | *bla*_CTX-universal_ – *ampC* – *aac*-(3)-IV – *aph(6)-la* – *aph(6)-Id* –  *qnrS – parC –* *tet*(A) – *tetB –* *sul1* – *sul*2 |
| C | C 2 | STa – STb – F4 | AMP – TIC – PIP – CFX – CXM – FOX – CTX – CPM– S – TE | *bla*_CTX-universal_ – *ampC* |
|  | C 6 | STa – STb – F4 | AMP – TIC – PIP –K – NE – APR – TE – SXT | *bla*_TEM_ – *ampC* – *aac*-(3)-II – *tet*(A) |
|  | C 7 | STa – STb – F4 | K – S – NE – APR – TE – SXT | *ampC* – *aac*-(3)-II – *tet*(A) |
| D | D 1 | F18 | FOX | *ampC* – *aac*-(3)-IV |
|  | D 2 | STb – LT – F18 | FOX – K – NE | *ampC* – *aac*-(3)-II |
|  | D 6 | F18 | FOX | *aph(6)-la* |
|  | D 8 | STb | NE | - |
|  | D 9  D 16 | F18  F18 | - | *ampC* |
|  | D 10 | STa – STb – F18 | K – NE – NA | *aac*-(3)-II – *parC* |
|  | D 13 | STb – LT – F18 | S – NA – TE – DO | *ampC* – *aph(6)-la* – *tet*(A) – *tetB* |
|  | D14 | F18 | FOX | *ampC* – *aac6’-Ib* |
|  | D 19 | F18 | SXT | *ampC* |
|  | D 21 | STb | FOX – S – NA – TE – DO | *ampC* – *parC* – *tet*B |
| E | E 3 | STb | AMP – TIC – CFX – S – NE – CTI – SXT | *bla*_TEM_ – *ampC* – *aph(6)-la* – *sul*1 – *sul*2 |
|  | E 4 | STb | AMP – TIC – PIP – S – TE – SXT | *bla*_TEM_ – *ampC* –*tet*(A) – *tet*B – *tet*M – *sul*2 |
|  | E 5 | STb | AMP – TIC – PIP – S – TE – SXT | *bla*_TEM_ – *ampC* – *aph(6)-la* – *sul*1 – *sul*2 |
|  | E 6 | STb | AMP – TIC – PIP – FOX – S – NE – SXT | *bla*_TEM_ – *ampC* – *aph(6)-la* – *sul*1 – *sul*2 |
|  | E 7 | STa – STb – F4 | AMP – TIC – PIP – CFX – CXM – FOX – CTX – CPM – CN – TOB – NE – TE | *bla*_TEM_ – *bla*_CTX-universal_ – *ampC* – *aac(6')-aph(2”)* – *aph(6)-Id* – *qnrS – parC* – *tet*(A) |
|  | E 9 | STb | AMP – TIC – S – SXT | *bla*_TEM_ – *ampC* – *aph(6)-la* – *sul*1 – *sul*2 |
|  | E 11 | STb | AMP – TIC – PIP – S – NE – TE – CTI – SXT | *bla*_TEM_ – *ampC* – *tet*(A) – *tet*M – *sul2* |
|  | E 12 | STb | AMP – AUG – TIC – PIP – S – CTI – SXT | *bla*_TEM_ – *ampC* – *aph(6)-la* – *mcr-2* – *sul*2 |
|  | E 21 | STb | AMP – TIC – PIP – S –TE – SXT | *bla*_TEM_ – *ampC* – *tet*(A) – *tet*M – *mcr*-2 – *sul*2 |
| F | F 14  F 16 | STb  STb | AMP – TIC – PIP – FOX | *bla*_TEM_ – *ampC* |
|  | F 17 | STb – LT – F18 | AMP – TIC – PIP – CXM – FOX – S – TE – DO – SXT | *ampC* – *aph(6)-la* – *sul*2 |
| G | G 1 | Sta – STb – F18 | AMP – AUG – TIC – PIP – CFX – CXM – FOX – CTX – CPM – CN – K – S – NE | *bla*_CTX-universal_ – *ampC* – *aac6’-Ib* – *ant*6 –*aph(6)-la* – *aph(6)-Id* – *tet*(A) – *mcr*-2 |
|  | G 4 | STb | AMP – TIC – PIP – S – TE – DO – SXT | *ampC* – *aac*-(3)-II – *aph(6)-la* – *tet*(A) – *sul*1 |
|  | G 7 | Sta – STb – F4 | AMP – TIC – PIP – S – NE – TE | *bla*_TEM_ – *ampC* – *aph(6)-la* |
|  | G 8 | Sta – STb – F18 | AMP – AUG – TIC – PIP – CFX – CXM – FOX – CTX – CPM – CN – K – S – NE – TE | *bla*_CTX-universal_ – *ampC* – *aac*-(3)-II – *aac(6')-aph(2”)* – *aac6’-Ib* – *ant*6 – *tet*(A) – *mcr-*2 |
|  | G 16 | Sta – STb – F18 | AMP – AUG – TIC – PIP – CFX – CXM – FOX – CTX – CPM – CN – K – S – NE – TE | *bla*_CTX-universal_ – *ampC* – *aac*-(3)-II – *aac(6')-aph(2”)* – *ant*6 – *aph(6)-la* – *tet*(A) – *mcr-*2 |
|  | G 19 | Sta – STb – F18 | AMP – AUG – TIC – PIP – CFX – CXM – FOX – CTX – CPM – CN – K – S – NE – TE | *bla*_CTX-universal_ – *ampC* – *aac*-(3)-II – *aac6’-Ib* – *ant*6 – *aph(6)-la* – *mcr-*2 |
|  | G 21 | Sta – STb – F18 | AMP – AUG – TIC – PIP – CFX – CXM – FOX – CTX – CPM – CN – K – S – NE – TE | *bla*_CTX-universal_ – *ampC* – *aac*-(3)-II – *aac6’-Ib* – *ant*6 – *tet*(A) |

AMP: ampicillin; AUG: amoxicillin + clavulanic acid; TIC: ticarcillin; PIP: piperacillin; CFX: cephalexin; CXM: cefuroxime; FOX: cefoxitin; CTX: cefotaxime; CPM: cefepime; CN: gentamicin; TOB: tobramycin; K: kanamycin; S: streptomycin; NE: neomycin; APR: apramycin; NA: nalidixic acid; ENR: enrofloxacin; MAR: marbofloxacin; TE: tetracycline; DO: doxycycline; CTI: colistin; SXT: Trimethoprim- sulfamethoxazole
